# Japanese Encephalitis—A Pathological and Clinical Perspective

**DOI:** 10.1371/journal.pntd.0000437

**Published:** 2009-09-29

**Authors:** Debapriya Ghosh, Anirban Basu

**Affiliations:** National Brain Research Centre, Manesar, Haryana, India; London School of Hygiene & Tropical Medicine, United Kingdom

## Abstract

Japanese encephalitis (JE) is the leading form of viral encephalitis in Asia. It is caused by the JE virus (JEV), which belongs to the family Flaviviridae. JEV is endemic to many parts of Asia, where periodic outbreaks take hundreds of lives. Despite the catastrophes it causes, JE has remained a tropical disease uncommon in the West. With rapid globalization and climatic shift, JEV has started to emerge in areas where the threat was previously unknown. Scientific evidence predicts that JEV will soon become a global pathogen and cause of worldwide pandemics. Although some research documents JEV pathogenesis and drug discovery, worldwide awareness of the need for extensive research to deal with JE is still lacking. This review focuses on the exigency of developing a worldwide effort to acknowledge the prime importance of performing an extensive study of this thus far neglected tropical viral disease. This review also outlines the pathogenesis, the scientific efforts channeled into develop a therapy, and the outlook for a possible future breakthrough addressing this killer disease.

## Introduction

A disturbing news item that was aired on news channels in August 2008 reported that the “kid killer” in Uttar Pradesh (UP) had struck again. Hospitals were flooded with patients, proper medical treatment was unavailable, and many people died [1].

This has been a very common report coming nearly every year from UP in northern India, which has become an epicenter for the killer disease Japanese encephalitis (JE). About 1,000 children die of this brain fever in UP every year. Statistical records show that more than 25,000 children have died of JE in the region since 1978, and the disease was started to be treated as a national health emergency in India in 2007 [2].

JE, one of the leading forms of viral encephalitis worldwide, is prevalent mostly in eastern and southern Asia, covering a region with a population of more than 3 billion. The disease affects mostly children. Around 30,000–50,000 cases of JE and up to 15,000 deaths are reported annually, although these statistics may be a gross underestimation because of inadequate surveillance and reporting. About 25%–30% of JE cases are fatal, and 50% result in permanent neuropsychiatric sequelae [3].

Generous efforts are being made by developed countries to deal with the grave conditions of JEV outbreaks in Asia [4],[5]. Unfortunately, the Western world is still underestimating the possibility of emergence of JE in the West [6]. JE is being treated as a rare and exotic disease and is not given priority in international public health programs [7]. This review outlines the pathogenesis of and the therapeutic prospects for JE and focuses on the exigency of developing a worldwide effort to acknowledge the prime importance of performing an extensive study of this neglected tropical disease.

## Ecology

JE virus (JEV) ecology has been widely studied. The virus exists in a zoonotic transmission cycle among mosquitoes, pigs, bats, and water birds belonging to the family Ardeidae (cattle egrets and pond herons). Humans become infected when bitten by an infected mosquito and are a dead-end host because of low viremia, preventing the virus from being transmitted further [8]. The major mosquito vectors of JEV vary in different geographic regions; the most common are those of the *Culex* genus [9],[10]. Pigs are the main contributors in the transmission cycle with respect to human infection, because these animals often stay close to human dwellings. Ardeid birds are important maintenance hosts. Recently, JEV antibodies were detected in bats, revealing that bats can be a part of the JEV transmission cycle [11]. Vertical transmission of JEV in mosquitoes probably explains the “overwintering” of virus between epidemics [10]. JEV infection in domestic animals and other vertebrate species such as equines does not result in high viremias; thus, they are not expected to transmit the virus to humans [12]. Amphibians and reptiles can be infected experimentally, but their role in overwintering and maintenance in the environment is not known [13].

## Origin and Spread

**Figure 1:**
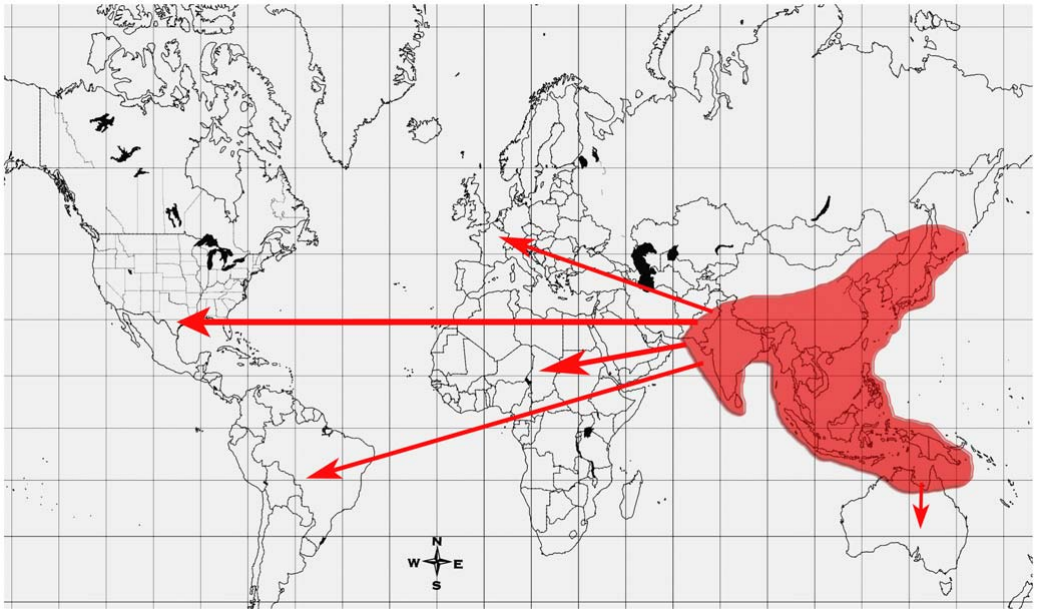
The global distribution of JEV genotypes. See [15]–[19]. The shaded areas indicate the present distribution of JEV, whereas the arrows indicate the areas where there is a high possibility of virus spread due to globalization and climatic change.

JEV was first isolated in Japan in 1935 but had been described there as early as 1870 [14]. JEV evolved from its ancestral flavivirus form into its present form in the Indonesia–Malaysia region. From there, it has spread to northward and westward directions in Asia and recently has been isolated in Australia [15]–[17]. To date, the *PrM* and *E protein* encoding genes have usually been used for the phylogenetic analysis of JEV [17]. Five genotypes of JEV are known at present. Different genotypes of JEV (associated with different virulence patterns) thrive in a particular climatic condition: genotypes IV (the oldest) and V are isolated in the tropical endemic region of Indonesia–Malaysia, whereas genotypes III and I are found in the epidemic regions [18],[19]. During the 1995 outbreak in Torres Strait, it was thought that JEV moved with the vagrant birds from island to island, from eastern Indonesia to New Guinea and Torres Strait. The subsequent spread of JEV into northern mainland Australia was suggested to be through the movement of infected mosquitoes blown by cyclonic winds [13],[20],[21]. The spread of flaviviruses such as JEV also seems to correlate with rapid globalization, the population explosion, and changes in global climatic condition due to industrialization and deforestation [22] (Figure 1).

According to a report published by the US Centers for Disease Control and Prevention [23], researchers believe that flaviviruses such as the West Nile virus (WNV) could have entered the West through infected travelers, unintentional introduction of infected birds, and/or virus-bearing mosquitoes. Thus, there is a high probability for such spread in the case of JEV [6].

## Clinical Manifestations

The manifestations of the disease depend on which part of the nervous system is affected and include early symptoms, such as nonspecific febrile illness, viz. diarrhea and rigor, followed by symptoms such as reduced levels of consciousness, seizures, headache, photophobia, and vomiting in the next stage [14]. In some cases, abnormal mental conditions may be observed. Later symptoms also include poliomyelitis-like flaccid paralysis [24] and parkinsonian syndrome, which manifests the classic description of JE—dull, flat, mask-like face with wide, unblinking eyes; tremor; generalized hypertonia; cogwheel rigidity; and other abnormalities in movement [14]. Severe encephalitis is associated with a higher frequency of seizures [8]. In fatal cases, patients ultimately slip into an acute coma. Various electroencephalographic abnormalities include the presence of alpha, theta, and delta coma, and epileptiform conditions [14],[25]. Symptoms of brainstem infection include changes in the respiratory pattern, flexor and extensor posturing, and abnormalities in the papillary and occulocephalic reflexes [14],[26]. In fatal cases of JEV, pathological changes are polymorphic and diffuse, involving various parts of the nervous system where the brain shows a severe degree of vascular congestion, microglial proliferation, formation of gliomesenchymal nodules, focal or confluent areas of cystic necrosis, cerebral edema, and transcompartmental shift [10],[27]. Many survivors of JE acquire neuropsychiatric sequelae with cognitive and language impairment, in which case the disease presents itself not only as a killer but also as a cause of an immense social and financial burden, especially for a developing country [3], [28]–[30].

## Pathogenesis

**Figure 2:**
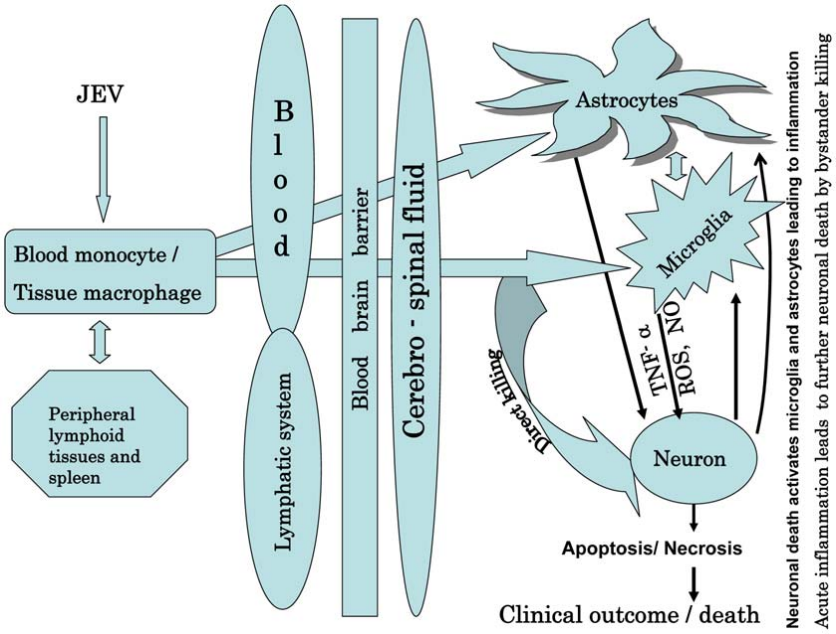
Events that lead to the establishment of JE pathogenesis.

A myriad of factors govern the severity of JEV pathogenesis (Figure 2). The failure of the host to produce antibodies against the virus is associated with an increased likelihood of the disease to turn lethal [31]. Crossing the blood–brain barrier is an important factor in the increased pathogenesis and clinical outcome of the neurotropic viral infection [32]. After entering the body through a mosquito bite, the virus reaches the central nervous system (CNS) via leukocytes (probably T lymphocytes), where JEV virions then bind to the endothelial surface of the CNS and are internalized by endocytosis [33]; however, it is still not clear whether macrophages and B lymphocytes can also harbor JEV. In other flaviviral infections, such as WNV, macrophages could serve as a reservoir, spreading the virus from the peripheral areas to the CNS [34]. Studies have shown that WNV is capable of entering the CNS through anterograde axonal transport [35]. Because both WNV and JEV belong to the same family of viruses [36]–[38], macrophage and axonal transport may play a critical role in JEV pathogenesis; however, convincing evidence is still lacking.

JE typically develops in patients after an incubation period of 5–15 days. It is possible that during this time, the virus resides and multiplies within host leukocytes, which act as carriers to the CNS. T lymphocytes and IgM play a major role in the recovery and clearance of the virus after infection [31]. A plausible therapy of clearing the virus load while in its incubation period in peripheral lymphatic tissues and spleen may actually prevent JEV pathogenesis. Besides neuronal cells, researchers have shown that astrocytes are also infected by JEV [18]. Astrocytes, being a component of the blood–brain barrier, may help in the transmission of JEV from peripheral tissues to the cerebrospinal fluid.

The molecular pathogenesis of JEV infection is still unclear. It is known that JEV causes neuronal cell death in two ways—direct neuronal killing [39], wherein viral multiplication within neuronal cells leads to cell death, and the indirect mode of killing, wherein massive inflammatory response causes an up-regulation of reactive oxygen species and cytokines such as tumor necrosis factor α (TNFα), which, in turn, causes neuronal death [40]. The key factor in indirect neuronal cell death during JE is the uncontrolled overactivation of microglia cells [40], which release proinflammatory cytokines such as interleukin 6 (IL-6), TNFα monocyte chemotactic protein 1 (MCP1), and RANTES (regulated upon activation, normal T cell expressed and secreted), promoting massive leukocyte migration and infiltration into the brain [41]. The production of interferon-γ–inducible protein 10 (IP-10) by activated astrocytes also contributes to the infiltration of natural killer cells and monocytes, among others [42]. Although nitric oxide (NO) plays an important role in inflammation during JE infection, NO itself is a very strong antimicrobial agent, and researchers have shown that it profoundly inhibits viral RNA synthesis, viral protein accumulation, and virus release from infected cells [43]. Thus, NO may play a crucial role in the innate immunity of the host and its ability to restrict the initial stages of JEV infection in the CNS. On the other hand, dysregulation of NO secretion may inflict a similar but toxic effect on uninfected host cells, thereby actually contributing to the pathogenesis of encephalitis.

Neuronal death by secreted TNF is mediated by the TNF receptor–associated death domain protein (TRADD) [44], which thereupon regulates a downstream apoptotic cascade in neurons. However, neuronal death also activates microglia and astrocytes; thus, the inflammatory cycle goes on.

The role of astrocytes in the pathogenesis of JE remains unknown. Research has shown that JEV infection differentially modulates the induction pattern of reactive oxygen species, IL-6, IL-8, IL-1, MCP-1, RANTES, and MIG secretions in different astrocytic cell lines [45]. Nevertheless, increased astroglial activation also has homeostatic implications as it enhances the ability of astrocytes to detoxify glutamate, inactivate free radicals, and produce neurotrophic factors, although it is not sufficient in conferring protection against JEV-mediated pathogenesis [46]. In vivo studies have reported that there is a progressive decline in the level of the anti-inflammatory cytokine IL10 following viral infection [47]. Thus, the intense cascade of proinflammatory mechanism takes over the homeostatic mechanism, leading to the pathogenesis of encephalitis.

Reports suggest that JEV infection affects neuronal progenitor cells (NPCs) in the subventricular zone and severely compromises their ability to proliferate. JEV infection does not result in the death of resilient NPCs, but the cycling ability of these cells is suppressed [48]. This arrested growth and proliferation of NPCs might be the cause of neurological consequences in children infected by JEV. Also, there are reports that JE can be transmitted transplacentally [49]–[51], by which means the virus could affect the normal neuronal development of the fetus.

## Diagnosis

Patients with JE present vivid signs of acute encephalitic syndrome. There are many possible causes of acute encephalitic syndrome; thus, laboratory confirmation is essential for the accurate diagnosis of JE, which is not a simple process because of the very low viremia. Diagnosis is therefore targeted toward the detection of antibodies in serum and cerebrospinal fluid. Cases of cross-reactivity of antibodies to other flaviviruses cause confusion in the diagnosis of JEV. The World Health Organization (WHO) has drawn up surveillance standards for the detection of JEV, recommended case definitions of JE, and set up criteria that are to be fulfilled to diagnose a case as JE [52]. IgM capture ELISA has been the most widely used diagnostic method for JE detection [53],[54]. At present, much advancement has been achieved in methods for the early detection of JEV; examples are the dipstick method [55], JEV-CheX [56], and reverse transcriptase PCR [57].

## Treatment

Interferon therapy has not met with great success [58]. A recent clinical trial of oral ribavarin administration was also not encouraging [59]. Naturally occurring compounds such as arctigenin, a phenylpropanoid dibenzylbutyrolactone lignan, and rosmarinic acid, a phenolic compound found in various Labiatae herbs, render protection to mice against JEV of the GP78 strain by markedly decreasing JEV-induced neuronal apoptosis, microglial activation, active caspase activity, and induction of proinflammatory mediators in the brains of the infected animals [60],[61].

A notable breakthrough in antiflaviviral drug research is the discovery of minocycline, a member of the broad-spectrum antibiotic tetracycline group, as an antiviral drug [62]. A significant piece of research on minocycline as an anti-JEV drug is an in vivo study [38] that showed that minocycline reduces neuronal apoptosis, microglial activation, active caspase activity, proinflammatory mediators, and viral titer markedly on the 9th day after infection. Another compound that has shown inhibition of JEV replication completely in vitro is an *N*-methylisatin-β-thiosemicarbazone derivative [63].

Glucosidase inhibitors of the endoplasmic reticulum, such as *N*-nonyl-deoxynojirimycin, which block the trimming step of N-linked glycosylation, have been shown to eliminate the production of several endoplasmic reticulum–budding viruses, including dengue type II (DEN-2) and JEV [64].

In an innovative study on mice using RNA interference, a single intracranial administration of lentivirus-delivered short hairpin RNA or lipid-complexed small interfering RNA (siRNA) before viral challenge or siRNA treatment after viral challenge was sufficient to provide protection against lethal encephalitis. Interestingly, when a cross-species conserved sequence of cd-loop coding viral envelope protein was targeted, encephalitis following both JEV and WNV challenge was avoided. This result clearly indicates that by careful drug design of the conserved target site, a single siRNA treatment could suppress viral infection across species, thereby augmenting the treatment of acute viral infections with overlapping clinical symptoms [65].

## Protection

One of the most important aspects of the prevention of JE spread is vector control. Paddy fields not only provide an ideal breeding ground for mosquitoes but also attract migratory birds, thereby helping the spread of the virus. Larvicides, insecticides, and ecofriendly methods such as using neem cakes and growing larvivorous fish are useful in controlling mosquitoes in paddy fields [9]. Vaccinating pigs is also a logical way to acquire protection against JEV by breaking the mosquito–pig–human transmission cycle [66]. Human vaccination is considered to be the most effective control measure for JEV. It is necessary to protect not only the residents of endemic regions but also others such as international tourists visiting rural Asia, where there is a growing risk of transmission of JEV to travelers, thus increasing the need for pretravel immunization with an effective vaccine product. Multiple vaccines exist to control JE, but all have limitations. Unfortunately, unlike smallpox, it is very difficult to eradicate JEV by vaccination, because human beings are actually a dead-end host of the virus.

The formalin-inactivated vaccine against JEV was produced from infected mouse brain–derived tissue soon after the virus was discovered. This type of vaccine became commercially available in Japan (as the Japanese Biken vaccine [JE-VAX]) and in Korea (as the Korean Green Cross vaccine) and was produced also in the United States [67]. This is the only WHO-recommended vaccine, but there are several concerns with its side effects [68],[69]. Moreover, the vaccine is expensive and requires multiple doses to maintain efficacy and immunity. This makes its use difficult for people in developing countries. Researchers have been trying to avoid a multidose requirement of the vaccine by using adjuvants such as biodegradable poly(gamma-glutamic acid) nanoparticles and aluminum [70].

In 1988, an inexpensive, live attenuated vaccine (SA 14-14-2) [71] was licensed by China. However, WHO does not approve it for human use because it is raised in primary kidney hamster cells. Initially, this vaccine was used almost exclusively in China and parts of Korea, but it is now widely used in the Indian subcontinent. The vaccine seems to be highly effective, and very few adverse effects have been observed [72]. The recently developed Vero cell–derived inactivated JE vaccine containing the purified, inactivated JEV strain SA 14-14-2 with aluminum hydroxide as adjuvant seems to be a promising candidate and has passed the Phase III randomized controlled trial [73].

Several efforts have been made to develop recombinant vaccines for JEV [3]. One study described a recombinant vaccinia virus expressing viral structural proteins. Later, a highly attenuated recombinant vaccinia virus, NYVAC [74], was used for JE vaccine development, and this proved quite successful [75]. Other strains tested include modified vaccinia virus Ankara strain [76],[77]. Later studies focused on safe replication-competent recombinant viruses such as avipoxviruses and ALVAC, which infect cells but do not replicate [74],[77],[78]. Again, in a research on peptide-based vaccine, Johnson grass mosaic virus coat protein was fused with JEV E protein by using recombinant DNA technology [79].

Researchers have also constructed the yellow fever virus to express JEV protein, the most promising recombinant vaccine under development. It is widely known as Chemeri-Vax-JE, in which the structural protein (PrM and E) of the JEV SA14-14-2 strain was inserted into YF17D [80]–[82]. In vivo studies have shown that passive immunization with monoclonal antibodies against different epitopes of JEV E protein protects against JEV-mediated cell death in mice [83].

Researchers have also tried DNA vaccination for JE in rhesus monkeys [84]. In one study, a single intramuscular injection of recombinant plasmid DNA containing JEV *PrM* and *E* genes induced protective immunity and prevented JE in mice and their progeny [85]. Again, investigators have used both intramuscular injection and a gene gun to deliver the plasmid gene for E protein and PrM protein [86]. DNA immunization with colloidal gold, construction of chimeric DNA vaccine vectors, and DNAzymes are the focus of some of the other recent promising studies [87]–[89].

## Future Perspectives

Flaviviral drug research is moving at an expeditious pace. We are hopeful that a dependable chemotherapeutic agent will soon be at hand to abrogate the flaviviral diseases. Fortunately, although JEV is presently under the radar of big, ambitious drug-design projects, owing to the conserved nature of the flaviviral proteins, research that has concentrated on WNV, hepatitis C virus (HCV), dengue virus (DENV), and others could be extrapolated to come up with a JEV-specific drug design.

Efforts are being channeled into the design of a future drug against the NS2B and NS3 nonstructural proteins of flaviviruses such as WNV. This design focuses on crucial physicochemical and biochemical properties of proteases by using crystallography-based models of NS3 substrate interaction compounds [90]. Scientists are also trying to target the flaviviral capping enzyme [91],[92].

The E and NS5 proteins of flaviviruses are also a very promising target for future drug designs. Research has elucidated the crystal structure of the enzymatically active domains of these proteins' inhibitor to understand its biochemical properties, subcellular localization, and regulation, thereby helping to design specific inhibitors that would alter the kinetics of these proteins and thus viral production [93]–[95].

Advances in our knowledge of the molecular biology of flaviviruses and construction of a flavivirus replicon system have paved the way for high-throughput screening of flaviviral inhibitors in an elaborate collaborative project using the mega-computing power of World Community Grid [96]. Researchers are conducting extensive calculations to identify new drug-like molecules against WNV, yellow fever virus, DENV, and HCV, by using binding calculations, mean-field molecular dynamics algorithms, Autodock virtual docking program, and CHARMM, a molecular dynamic program. The scale and depth of this project carry enough promise for a significant breakthrough in flaviviral drug research.

Finally, studies revealed that different classes of compounds such as flavonoids, alkaloids, polysaccharides, thiophenes, terpenoids, lectins, and lignans, isolated from various plants, have different antiviral properties and target viral inhibition [97]. More studies are needed to exploit the potential that natural products may possess to be developed into anti-JEV drugs.

## Conclusion

With cutting-edge technologies of biomedical science at hand, the future bears hope for a breakthrough in JEV therapy. Unfortunately, only a handful of laboratories in developed countries are actually involved in any rigorous study involving JEV. If not for its similarity with the other flaviviruses such as WNV, DENV, and HCV, its present research status could have been worse. The scientific communities all over the world should be more concerned about the recent emergence of JE in areas of the world where it was previously unknown. A considerable percentage of JEV outbreaks occur in developing countries. Therefore, it is the responsibility of the scientific communities, federal governments, and WHO to find drugs that could reach the unprivileged masses. Only then will our scientific efforts be a success.
